# Evaluation of prognostic factors associated with antrochoanal polyp recurrence: Case series and literature review

**DOI:** 10.1016/j.ijscr.2025.111744

**Published:** 2025-07-31

**Authors:** B. Saout Arrih, W. Bijou, Y. Oukessou, S. Rouadi, R. Abada, M. Mahtar

**Affiliations:** Department of Otolaryngology, Head and Neck Surgery, Ibn Rochd University Hospital, Faculty of Medicine and Pharmacy, Hassan II University, Casablanca, Morocco

**Keywords:** Antrochoanal polyp, Prognostic, Factors, Recurrence, Caldwell Luc surgery, Case series

## Abstract

**Introduction and importance:**

Despite being non-neoplastic and usually unilateral, antro-choanal polyps have a notable tendency to recur following surgical treatment, particularly when incomplete removal of the polyp's antral portion occurs. Understanding the mechanisms and risk factors associated with ACP recurrence is crucial for optimizing treatment strategies and minimizing the likelihood of relapse. The main objective of this study was to identify and analyze the prognostic factors associated with recurrence of this benign but potentially recurrent pathology.

**Methods:**

This is a retrospective study of a series of 27 patients operated for an antrochoanal polyp (ACP). The study was carried out over a five-year period, from January 2, 2017 to July 5, 2024. All patients, regardless of age or sex, who underwent surgery for an antrochoanal polyp during this period were included in the study.

**Results:**

A total of 21 patients had no recurrence during follow-up. Of these, 14 patients (67 %) underwent endoscopic nasal endoscopy with meatotomy. However, 7 patients (33 %) underwent Caldwell-Luc (CWL) approach associated with endoscopic surgery. Recurrence was noted in 6 patients (22.22 %). The mean time to recurrence was 22 months, of which 4 (66 %) recurred before 16 months. We found that 50 % of patients who recurred (3 cases) were aged between 13 and 36, 17 % were under 13 (1 case) and 33 % were over 36 (2 cases). Recurrences were equally distributed between the genders, with a sex ratio of 1. All patients who had recurrence were of urban origin. Three patients, i.e., 50 % of those who recurred, had a rhino-sinus history. All patients who had relapsed underwent exclusive endonasal endoscopy with middle meatotomy for removal of the sinus component of ACP. Also, all patients had a tumor size greater than 5 cm. Following the results of our analytical study between the relapsed group (6patients) and the non-relapsed group (21patients), there were three prognostic factors found to be significantly associated with relapse with a p value <0.05. Associated endonasal pathology (p = 0.040) increases risk of recurrence. Tumor size ≥5 cm (p = 0.005). Incomplete surgical excision (p = 0.020).

**Conclusion:**

Relapse of antrochoanal polyps is often due to incomplete surgical removal and persistent inflammation. Proper identification of recurrence risk factors and the use of complete endoscopic excision with wide maxillary antrostomy are essential to reduce recurrence. Long-term follow-up is crucial for early detection and effective management.

## Highlights

• Antrochoanal polyp inserts into the maxillary sinus, exiting into the nasal cavity and into the nasopharynx.
• Treatment is exclusively surgical. It consists of complete removal of the polyp, including its implantation base in the maxillary sinus, to limit the risk of recurrence.
• Antrochoanal polyps present a significant risk of postoperative recurrence.
• Relapse depends on several, lesion size, pedicle location, nasosinus comorbidities, diagnostic and therapeutic delay, and above all the quality of surgical excision.

## 1 Introduction

The antrochoanal polyp (ACP), also known as Killian's polyp, is a benign, non-atopic, unilateral, solitary tumor that originates in the maxillary sinus and extends into the choana [1]. It inserts into the maxillary sinus and around the sinus ostium, exiting into the nasal cavity and via the choana into the nasopharynx. It is an uncommon tumor, occurring mainly in adolescents and young adults, in both males and females, and its etiopathogenesis is unclear [2].

The polyp does not usually erode or invade adjacent tissues. Diagnosis is based on clinical symptoms, mainly nasal obstruction and unilateral rhinorrhea. Nasosinus endoscopy helps to orientate the diagnosis by showing the typical appearance of an ACP. CT scan of the paranasal sinuses supports the diagnosis [1,2].

Treatment is exclusively surgical. It consists of complete removal of the polyp, including its implantation base in the maxillary sinus, to limit the risk of recurrence [[1], [2], [3]].

The main objective of the following study is to describe the main prognostic factors for recurrence of antrochoanal polyps after surgical treatment.

This case series has been reported in line with the PROCESS guidelines [20].

## 2 Materials and methods

This is a univariate retrospective study of a series of 27 patients operated for an antrochoanal polyp (ACP). The main objective of this study was to identify and analyze the prognostic factors associated with recurrence of this benign but potentially recurrent pathology.

The study was carried out over a five-year period, from January 2, 2017 to July 5, 2024.

All patients, regardless of age or sex, who underwent endoscopic and/or Caldwell Luc surgery for an antrochoanal polyp during this period were included in the study. The surgery was performed by ENT specialist.

From patient medical records, we collected demographic and epidemiological data (age, gender, medical history, etc.), clinical data (symptomatology, endoscopic examination), paraclinical data (imaging), anatomopathological data, therapeutic modalities (surgical technique, medical treatment) and evolutionary data (postoperative follow-up, recurrence, possible complications).

Statistical analysis was carried out using SPSS (Statistical Package for the Social Sciences) software. Statistical significance was defined as a p-value <0.05, with a 95 % confidence interval (CI).

This case series has been reported in line with the PROCESS Guideline [20].

## 3 Results

The average age of our patients was 27, with extremes ranging from 6 to 62 years old, and a peak in the age range 13 to 36 years, and 40.7 % in the age range over 36 years. There were 13 men (48.1 %) and 14 women (51.9 %), with a sex ratio of 0.93 (M/F). We noted that 23 of our patients were of urban origin, i.e., 85.2 %, and 4 of rural origin, i.e. 14.8 %.

Twenty-four patients had no particular pathological history, 1 patient had a history of allergic rhinitis (3.7 %), and 1 patient had chronic rhinosinusitis (3.7 %). Four patients underwent surgery for ACP, i.e., 14.8 %. No family history of rhino-sinusitis was reported.

Delays to consultation ranged from 1 month to 15 years, with the highest frequency at over 3 months (96.3 %) and only 3.7 % of patients consulting within 3 months. Nasal obstruction was constant in all patients (100 %). It was unilateral on the right in 8 cases (29.6 %), unilateral on the left in 15 cases (55.5 %) and bilateral in 4 cases (14.8 %). Rhinorrhea was found in 14 patients (51.87 %).

On rhinoscopy examination, the mucosa was inflammatory in all cases, nasal secretions were purulent in 2 cases (7.4 %) and watery in 25 cases (92.6 %). Septal deviation was present in 5 cases (18.51 %) and hypertrophy of the inferior turbinate in 1 case (3.70 %) (Table 1).

**Table 1:** Results of nasal endoscopy.

Physical symptoms	Number of cases	Percentage
Nasal fossa polyp	27	100 %
Inflammatory mucosa	27	100 %
Presence of associated nasal pathology	6	22,22 %
Septal deviation	5	18,51 %
Watery secretions	25	92,60 %
Purulent secretions	2	7,40 %
Hypertrophy of the inferior turbinate	1	3,70 %

**Fig. 1:**
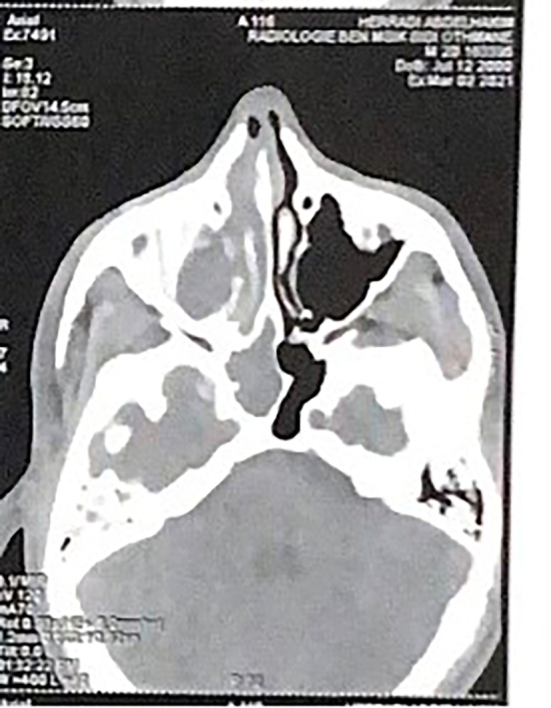
CT axial section showing an extensive right antrochoanal polyp.

In our series, CT showed non-extensive ACP in 2 cases (7.4 %). Extensive ACP in 25 cases (92.6 %), extending beyond the maxillary sinus and middle meatus, with filling of the ethmoidal cells in 14 cases (51.85 %), thickening of the sphenoidal mucosa in 10 cases (37, 03 %), thickening of the frontal mucosa in 4 cases (5.92 %). Hypertrophy of the inferior turbinate was noted in 4 cases (5.92 %), homolateral septal deviation in 7 cases (25.92 %), no bone lysis noted (Fig. 1).

ACP was unilateral in 26 cases, involving the left side in 16 cases (59.26 %) and the right side in 10 cases (37.03 %). Bilateral in 1 case (3.7 %).

All patients underwent endoscopically guided endonasal surgery. Lesion excision consisted of excision of the endonasal and endo-sinusal components. 20 patients (74 %) were treated exclusively by endonasal endoscopic surgery with middle meatotomy. Seven (26 %) patients underwent a Caldwell-Luc surgical approach associated with endoscopic surgery.

In our series, the base of implantation was confirmed in 6 cases (22 %), while the rest were uncertain (Table 2).

**Table 2:** Location of antrochoanal polyp implantation pedicle.

Site of ACP implantation pedicle	Number of cases
Unidentified	21
Anterior wall of the maxillary sinus	2
Lateral wall of the maxillary sinus	2
Inferior wall of the maxillary sinus	1
Posterior wall of the maxillary sinus	1

All patients received nasal swabs for 48 h under antibiotic cover, analgesic prescription, nasal cavities washed several times a day with saline solution for 4 weeks, combined with local corticosteroid spray for 3 months. Hospital stay did not exceed two days. Postoperative follow-up was rigorous.

A total of 21 patients had no recurrence during follow-up. Of these, 14 patients (67 %) underwent endoscopic nasal endoscopy with meatotomy. However, 7 patients (33 %) underwent Caldwell-Luc (CWL) approach associated with endoscopic surgery. The main characteristics of these patients are shown in Table 3.

**Table 3:** Epidemiological, clinical, surgical and monitoring data on patients who have not relapsed.

Variables		Number of cases with percentage (%)
Age	<13 years	4 (19 %)
	13 years–36 years	8 (38 %)
	>36 years	9 (43 %)
Gender	Men	10 (48 %)
	Women	11 (52 %)
Consultation period	<3 months	0 (0 %)
	>3 months	21 (100 %)
Residence	Urban	17 (81 %)
	Rural	4 (19 %)
Polyp extension	Yes	20 (95 %)
	Non	1 (5 %)
Type of surgery	Endonasal endoscopy	14 (67 %)
	Combined endonasal and CWL approach	7 (33 %)
Complete excision	Yes	11 (52 %)
	No	10 (48 %)
Tumor size	<5 cm	14 (67 %)
	>5 cm	7 (33 %)
Regular check-ups	Yes	21 (100 %)
	No	0 (0 %)

**Fig. 2:**
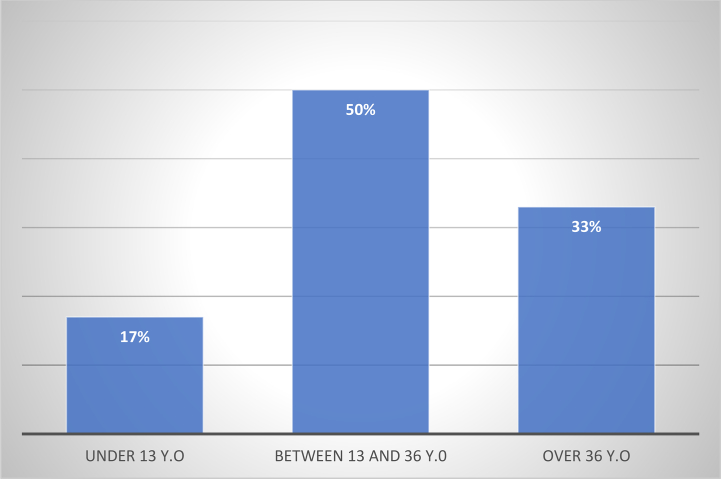
Distribution of recurrences by age group.

Recurrence was noted in 6 patients (22.22 %). The mean time to recurrence was 22 months, of which 4 (66 %) recurred before 16 months. We found that 50 % of patients who recurred (3 cases) were aged between 13 and 36, 17 % were under 13 (1 case) and 33 % were over 36 (2 cases). Recurrences were equally distributed between the genders, with a sex ratio of 1. All patients who had recurrence were of urban origin (Fig. 2).

**Fig. 3:**
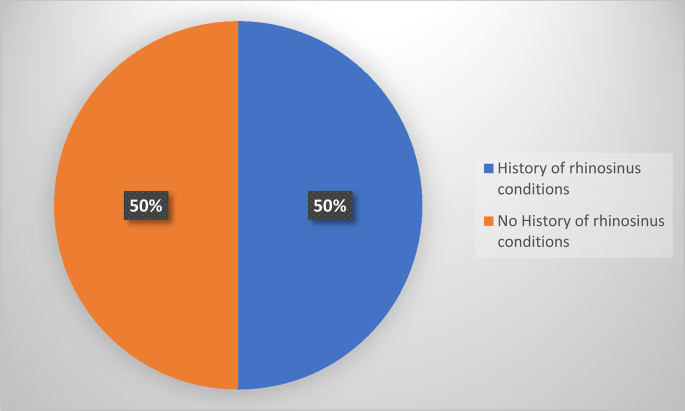
Distribution of recurrences according to medical history.

Three patients, i.e., 50 % of those who recurred, had a rhino-sinus history, including one patient with allergic rhinitis, and 1 patient with a previous surgery for an antrochoanal polyp. Five patients, or 83 % of those who had relapsed, were seen after more than 3 months, versus 1 patient, or 17 %, who was seen before 3 months. Four patients (67 %) who had relapsed had an extensive polyp, versus two patients (33 %) who had a non-extensive polyp on endoscopic examination (Fig. 3).

**Fig. 4:**
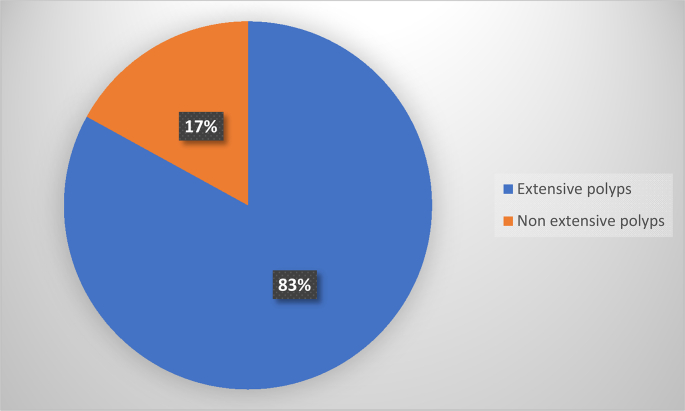
Distribution of recurrences according to the extent of the polyp on endoscopic examination.

All patients who had relapsed underwent exclusive endonasal endoscopy with middle meatotomy for removal of the sinus component of ACP. Also, all patients had a tumor size greater than 5 cm. It was also observed that for all patients who had relapsed, the base of implantation had not been determined at the time of surgical excision (Fig. 4).

Following the results of our analytical study between the relapsed group (6patients) and the non-relapsed group (21patients), there were three prognostic factors found to be significantly associated with relapse with a p value <0.05. Associated endonasal pathology (deviated nasal septum, allergic rhinitis, chronic rhinosinusitis, concha bullosa) (p = 0.040) increases risk of recurrence. Tumor size ≥5 cm (p = 0.005). Incomplete surgical excision (anatomical constraints (e.g., difficult access, poor visualization, or bleeding) or intraoperative uncertainties) (p = 0.020). Age, sex, place of residence, time to consultation and medical history had no statistically demonstrated effect on recurrence (Table 4).

**Table 4:** Prognostic factors according to our study.

Variable	Recurrence (n = 6)	No recurrence (n = 21)	p-Value
Age <13 years	1	4	1.0
Age 13–36 years	3	8	1.0
Age >36 years	2	9	0.9604
Male (M)	3	10	1.0
Female (F)	3	11	1.0
Urban residence	6	17	0.9463
Rural residence	0	4	0.6052
Time to consultation ≥3 months	5	21	0.7804
History of allergic rhinitis	1	0	0.527
History of rhino-sinusitis	1	0	0.527
Operated for ACP	1	3	1.0
Associated endo-nasal pathology (deviated nasal septum, allergic rhinitis, chronic rhinosinusitis, concha bullosa)	4	2	**0.040**
Extensive polyp on CT	5	20	0.866
Tumoral size ≥5 cm	6	7	**0.005**
Exclusive endoscopic surgery	6	14	0.647
Combined Caldwell-Luc approach	0	7	0.304
Incomplete surgical excision	6	9	**0.020**
Evidence of implant base	0	6	0.379
Postoperative monitoring	6	21	1.0

Statistical significance is defined by a P value of less than 0.05.

## 4 Discussion

In our study, the recurrence rate for antrochoanal polyps was 22.22 %, with a mean time to recurrence of 22 months.

In comparison, the study by Cook et al. of 33 cases reported a recurrence rate of 28 % [4]. Ozdek et al. studied 10 children and found a recurrence rate of 20 %, noting that recurrence was more frequent in younger children and those who had undergone only a middle meatotomy [5]. In another study, Bozzo et al. reported a recurrence rate of 8.6 % in 23 cases after endoscopic surgery alone, suggesting that complete excision of the polyp reduces recurrence [6]. Lee and Huang, in a study of 26 children, found a recurrence rate of 11.5 %, emphasizing that incomplete removal could account for these recurrences [7]. The study by Adnane et al., involving 31 pediatric cases, reported a recurrence rate of 16.1 % with a mean time to recurrence of 18 months, showing that the addition of a Caldwell-Luc surgery enabled more complete excision and reduced the risk of recurrence [8].

A study of 23 patients by Bozzo et al. reported two recurrences, both in children. The proximity of critical structures (orbit, lacrimal duct, dental buds) makes complete excision of the polyp more difficult, explaining the higher recurrence rate in this population [6]. Similarly, an Italian study by Frosini et al. involving 200 patients observed relapses only in children under 7 initially treated with simple polypectomy [9]. This trend could be explained by the incomplete development of naso-sinusal structures and the narrowness of the nasal cavity, making surgical access more complex, thus increasing the risk of residual [7]. Furthermore, surgeons often adopt less invasive approaches to preserve facial growth and minimize complications, which can lead to incomplete resection [10]. However, in our series, age was not a significant factor in recurrence (p = 0.869), which agrees with an Egyptian study that analyzed 22 patients aged 12 to 45 and found no relationship between age and recurrence of ACP [11].

A Taiwanese study by Lee et al. evaluated endoscopic treatment of antrochoanal polyps in children, and found a recurrence rate only in patients with chronic rhinosinusitis. The study shows that this pathology is more frequent in children over 10 years of age, and appears to be a prognostic factor for recurrence. Statistical analysis (p = 0.038) confirmed a significant association between age, chronic sinusitis and ACP recurrence, underlining the importance of appropriate management of nasosinus pathologies to reduce the risk of recurrence. Moreover, persistent inflammation and mucosal edema complicated the distinction between healthy tissue and polyp, making excision more difficult and favoring recurrence. Similarly, the study by Saafan et al. reported two cases of recurrence associated with rhinosinusitis, suggesting that chronic inflammation may be a contributing factor. Persistent rhinosinusitis impairs sinus drainage and ventilation, creating an environment conducive to polyp recurrence [7]. However, Hammouda et al. conclude that associated nasosinus pathologies (septal deviation, turbinate hypertrophy) do not significantly influence recurrence in their series and that the key to avoiding recurrence lies in surgical technique [12].

The size or volume of the antrochoanal polyp can increase the risk of recurrence by making complete excision more difficult, particularly for voluminous polyps that leave microscopic residues. In addition, a large ACP may cause prolonged obstruction of the ostium, leading to chronic local inflammation and mucosal alteration, thus favoring polyp regrowth. Studies converge on one essential point; it is above all the quality of surgical excision that determines prognosis [[12], [13], [13], [14]].

Notably, pediatric patients more frequently present with extensive CT stages, implying larger lesion volume and delayed diagnosis, potentially leading to stronger tissue remodeling stimuli. Additionally, histologic studies have demonstrated prominent edema and intramural cysts within ACPs that correlate with stromal expansion and remodeling under pressure forces. Recent immunohistochemical data highlight a neutrophil-predominant inflammatory profile supportive of active remodeling and angiogenic activity distinct from eosinophilic CRS [21].

Complete removal of the polyp, including its insertion pedicle in the maxillary sinus, is essential to prevent recurrence. Several studies have highlighted the influence of polyp location on recurrence rate, but no significant association has been found. A recent Italian study in 2020 on 82 patients showed that polyps located on the lateral wall recurred in 33 % of cases, whereas no recurrence was observed for those on the posterior wall [14]. According to Saafan et al., the risk of recurrence increases when ablation is incomplete, particularly when the pedicle is located on the anterior or inferior wall of the maxillary sinus [11]. Similarly, Hammouda et al. reported a 12 % recurrence rate associated with a lateral origin of the pedicle, difficult to visualize without angled instruments [12]. Finally, Lee and Huang observed a higher rate of recurrence for ACP located on the lateral or anterior walls, areas less accessible by endoscopy alone [7] (Table 5).

**Table 5:** Comparison of recurrences considering the site of ACP attachment for the standard surgical approach according to the study by Al-Balas [19].

Attachment site	Number of patients	Standard approach	Recurrence (%)
Unidentified	34	19	3 (15.8)
Anterior wall	10	5	1 (20)
Posterior wall	4	4	0
Lateral wall	8	6	2 **(33****)**
Inferior wall	26	15	3 (20)
**Total**	**82**	**49**	**9 (18.4** **%)**

Incomplete excision is the factor most frequently associated with recurrence of antrochoanal polyps, and surgical technique has a significant influence on prognosis in terms of recurrence rate, morbidity and postoperative quality of life. Originally, the Caldwell-Luc surgery was considered the gold standard, but its risks for maxillary growth in children limit its use [15].

Franche et al. note a recurrence rate of 6.9 % with endoscopic approach alone [16], while Freitas et al. and Ozdek et al. observe recurrence rates of 12.5 % and 20 % respectively [17]. Pagella et al. underline the efficacy of the combined endoscopic and CWL approach, 0 % recurrence, vs. 22 % for the standard endoscopic technique [3] (Table 6).

**Table 6:** Postoperative complications of ACP according to surgical technique [18].

Complication	Endoscopic surgery (n = 19)	Mini-Caldwell + endoscopic surgery (n = 21)	p value (post-op ESS vs. Mini-Caldwell)
Synechia	7 (36.8 %)	5 (23.8 %)	>0.05
Hemorrhage	5 (26.3 %)	4 (19 %)	>0.05
Ostial stenosis	3 (15.7 %)	3 (14.2 %)	>0.05
Relapse	4 (21.1 %)	0	**0.042** (<0.05)

Statistical significance is defined by a p-value < 0.05.

A prospective study of 60 patients by Abdelraouf et al. compared the efficacy of medial meatotomy alone and the prelacrimal recess's approach assisted by medial meatotomy, clearly demonstrating that the prelacrimal recess approach combined with medial meatotomy offers significant advantages over medial meatotomy alone, resulting in a zero-recurrence rate versus 16.7 % for medial meatotomy alone. This is due to the improved visualization and greater access to the maxillary sinus afforded by this approach, enabling more complete excision of the polyp and its pedicle, particularly in the case of anterior wall lesions [13] (Table 7).

**Table 7:** Differences between middle meatotomy (MM) alone and the prelacrymal recess approach (ARPL) assisted by a middle meatotomy, based on the results of the study by E. Abdelraouf [13]

Criteria	MM only (Group A)	ARPL + MM (Group B)	Statistical signification (p-value)
Operating time (min)	25,2 ± 3,5	37,7 ± 4,2	**<0,001**
Recurrence rate	16,7 % (5/30 cases)	0 % (0/30 cases)	**0,03**
Postoperative pain	Less intense (score ≤ 4)	More intense (score ≤ 6)	**0,006 at 4 weeks**
Complications			
- Synechia	3,3 % (1/30)	16,7 % (5/30)	0,1 (non-significant)
- Nasal obstruction	10 % (3/30)	20 % (6/30)	0,3 (non-significant)
- Epiphora (watery eyes)	0 %	6,7 % (2/30, grade 1*)	
Post-operative scabs	Less frequent	More frequent (1–3 months)	**0,01 at 1 month**/**0,04 at 3 months**

Statistical significance is defined by a p-value < 0.05.

In our study, incomplete excision was found to be significantly associated with recurrence, with a P = 0.02 value.

When the site of origin is anterior or lateral within the maxillary sinus, visualization and access become more challenging. In such cases, advanced endoscopic techniques are warranted. One such method is the prelacrimal approach, which allows access to the anterior maxillary wall without disrupting the inferior turbinate or nasolacrimal duct [22].

A further refinement of this technique is the Dissection Around the Lacrimal-Maxillary Attachment (DALMA) approach, introduced by Omura et al. at Jikei University, Tokyo. This method provides excellent visualization of the anterior, lateral, and inferior maxillary sinus walls while preserving critical nasal structures. The DALMA technique is particularly useful in cases where the pedicle arises from difficult-to-access areas and offers a minimally invasive alternative to external or more aggressive surgical routes [23].

## 5 Conclusion

Although benign, antrochoanal polyps present a significant risk of postoperative recurrence, depending on various factors, lesion size, pedicle location, nasosinus comorbidities, diagnostic and therapeutic delay, and above all the quality of surgical excision.

Curative treatment is exclusively surgical, with endoscopic endonasal surgery as the gold standard. For complex or recurrent forms, two complementary approaches to the standard endoscopic surgery stand out, the Caldwell-Luc technique, providing direct access to the maxillary sinus, and the prelacrymal recess transnasal route, offering excellent access to the anterior wall of the sinus leading to a low recurrence rate.

Continuous follow-up by nasal endoscopy is essential for early detection of any recurrence. An individualized therapeutic strategy, adapted to the specificities of the lesion and the patient, is the most effective approach for reducing the risk of recurrence.

## CRediT authorship contribution statement

Dr. Saout Arrih Badr: Corresponding author and writing the paper
Pr. Walid Bijou: Study concept and writing the paper
Pr. Oukessou Youssef: Study concept and correction of the paper
Pr. Abada Reda: Study concept and correction of the paper
Pr. Rouadi Sami: Study concept and correction of the paper
Pr. Mahtar Mohamed: Study concept and correction of the paper.

## Consent

Written informed consent was obtained from the patients or the patient's parents for publication of this case report and accompanying images. A copy of the written consent is available for review by the Editor-in-Chief of this journal on request.

## Ethical approval

Obtained

## Guarantor

Saout Arrih Badr.

## Research registration number

N/a.

## Provenance and peer review

Not commissioned, externally peer-reviewed.

## Funding

None.

## Declaration of competing interest

None.
